# Supplementary material for: Fully Automated Deep Learning-Based Pipeline for Evans Index Measurement from Raw 3D MRI
Source: medRxiv. 2025 Dec 2:2025.11.30.25341302. Preprint. [Version 1] doi: 10.64898/2025.11.30.25341302 (PMC12706619; doi:10.64898/2025.11.30.25341302)
Supplement: Supplement 2 [file media-2.docx]

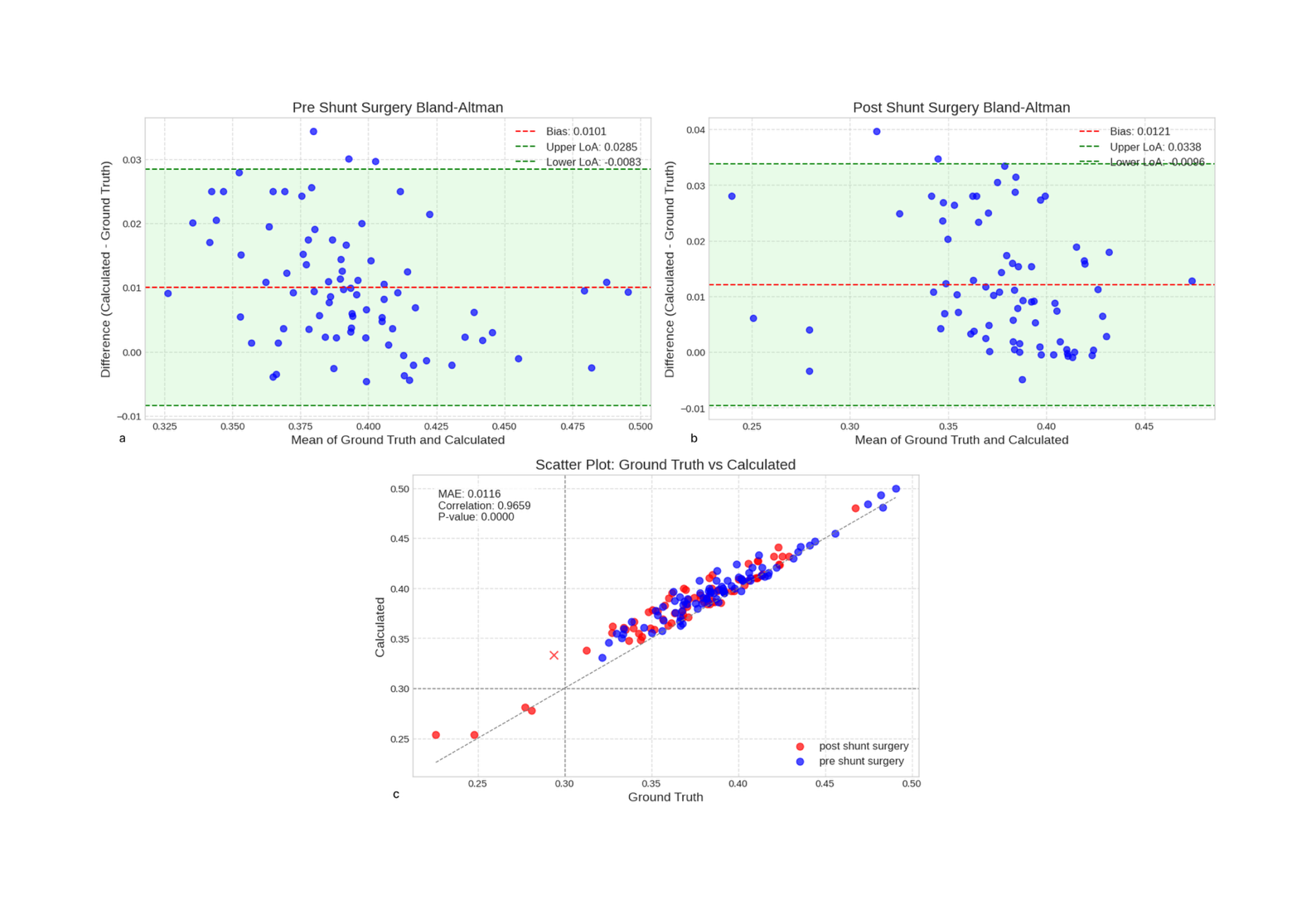


**Supplementary Figure 1.** Performance evaluation of automated Evans Index calculation using DLMUSE for lateral ventricle segmentation. (a–b) Bland–Altman plots comparing automated Evans Index measurements with expert annotations for pre-shunt (a) and post-shunt (b) scans. (c) Scatter plot showing correlation between automated and expert measurements, with pre-shunt scans in blue and post-shunt scans in red. Point(s) marked with X represent misclassified data at a 0.30 threshold.
